# LGBTQs and LAW’S Violence Within a Heteronormative Landscape

**DOI:** 10.3389/fsoc.2021.564028

**Published:** 2021-03-09

**Authors:** Anthony Donnelly-Drummond

**Affiliations:** Department of Social Sciences, Leeds Beckett University, Leeds, United Kingdom

**Keywords:** law’s violence, LGBTQs, peripheral inclusion, equality Act 2010, equality Act NI 2006

## Abstract

This article raises concerns as to the way in which specific exemptions within equality law across the United Kingdom may be viewed as a form of violence against LGBTQs.^1^ The overarching concept of heteronormativity and its impact is aired. The possible impact of exemptions for religious organisations to legally discriminate on grounds of sexual orientation under Northern Ireland’s Equality Act 2006 acting as a catalyst for the Asher’s cake incident, is reviewed. Similarly, the effect of exemptions under the Equality Act 2010 feasibly lending support for protestors against inclusion of LGBTQ issues in a Birmingham school is made clear. Thereafter the invisibility of LGBTQs within curricula is raised alongside the legacy of section 28. Overall, the article raises the spectre that specific aspects of equality legislation, designed to protect individuals on a range of grounds, may be perceived as not only flawed but inherently violent due to the dehumanizing impact on LGBTQ people. In addition, concerns are raised that against this background, within the United Kingdom there has been a failure to educate all students as to contemporary issues of diversity, potentially harming their development as citizens in our diverse contemporary society.

## Introduction

The focus of this paper is to raise concerns as to equality, diversity and cohesion and especially, the health and well-being of LGBTQs within all areas of education. This is accomplished by arguing that across the United Kingdom, particular aspects of equality law, its purpose being to protect individuals from discrimination on the grounds of their protected characteristics^2^ can, in two specific circumstances reviewed here (both events underscored by exemptions within equality law designed to protect and promote religious dogma) be viewed (at the very least) as psychological/verbal violence against LGBTQ people.^3^ Within this apparent heteronormative landscape it is demonstrated below how the human dignity of LGBTQs is diminished.

In the first instance the concept of heteronormativity is raised briefly to underscore its overall impact on LGBTQ people. Thereafter the spectre of law’s violence is critiqued relative to equality legislation. The potential influence of exemptions related to the Equality Act Northern Ireland underscored by the infamous Asher’s cake row in that jurisdiction is redressed whilst links are also made between similar exemptions under equality law in Britain and the long running protest at inclusive education outside a Birmingham school in 2019. Prior to concluding this work the issue of LGBTQ inclusion within curricula (including that of higher education) is assessed in some detail to underscore the situation for this community to date.

### Heteronormativity

A report by Syracuse University (2004: iv) stated that “heteronormativity” refers to “an ideology based on definitions of what it means to be a woman or a man that exclude and discriminate against a significant minority population.” Therefore wherever this system of oppression is identified within the education system “..there remains an onus on willing educators to disrupt and undo [it]” (Adams 2004 in Syracuse University, 2004: iv). As will become evident, the ideology of heteronormativity is echoed in exemptions to equality legislation, and, apparent within the education system per se. Yet it is argued below that whilst, to varying degrees, all law may be considered violent in its consequences the exemptions impacting LGBTQs under equality laws across the United Kingdom related to religious dogma sit uncomfortably with the main ethos of such legislation: to protect from discrimination as opposed to embedding some discrimination within such laws.^4^ In the first instance then there is a need to interrogate what is meant by the violence inherent within law.

### LAW’S Violence

Before discussing issues concerning the situation of LGBT students within society, including the education system, it is pertinent to raise concerns at what is perceived here to be the violence pertaining to exemptions within equality legislation across the United Kingdom and, to reflect on the signification of this for those who may like to feel not only right [teous] but likeable if they openly discriminate against LGBTQs. Hay (1993) believes that all law is violent. The violence of law need not be considered purely as physical or psychological when for example a prisoner is incarcerated. Whilst jail can be experienced physically and emotionally, the poverty and deprivation suffered by offspring as a consequence of parents being fined (often females) for example, may also be considered as violence when a mother cannot afford to care properly for her children when fined (or jailed) for instance, as a result of not being able to afford a tv licence (Gil, 2019). Nevertheless, within Britain under the Equality Act 2010 LGBTQs are protected from discrimination in access to goods and services.^5^. Yet, as made clear below, within that Act, and the almost identical one preceding it in Northern Ireland, discrimination (a form of psychological violence, if not physical dependent upon the longer term impact of it) against LGBTQs is also *legally* permitted in certain cases.

Northern Ireland's equality legislation was enacted in 2006 and contains exemptions permitting discrimination against Queer people. On the ground of sexual orientation, under the Northern Ireland Statutory Rules 2006 No. 439 of the Equality Act 2006, within the remit of “Organisations relating to religion or belief” it is stated that:

(5) Paragraphs (3) and (4) permit a restriction [essentially, permitting discrimination]^6^ only if imposed —
(1) if it is necessary to comply with the doctrine of the organization; or
(2)so as to avoid conflicting with the strongly held religious convictions of a significant number of the religions followers.^7^

Across Britain almost identical exemptions apply under the Equality Act 2010. As stated within a briefing by the Universities and College Union (2019) there are specific exemptions within the Equality Act 2010 “for religious organisations (schedule 23) 4 in relation to sexual orientation.” More specifically, as revealed by the Equality and Human Rights Commission (2016) an exemption is permissible allowing:

…a non-commercial religion or belief organization to impose restrictions in relation to sexual orientation where this is necessary to comply with its doctrine, or to avoid conflict with the strongly held convictions of a significant number of members of the religion or belief. (*ibid:* 41).^8^

Likewise the Citizens Advice (2020) website blog: “Religious organisations and charities: when discrimination is allowed in the provision of goods or services” states that “…there are some situations in which discrimination is allowed” permitting “…exclusion from participation in certain activities or services … [to] restrict membership of the organization [and] restrict the use of its premises”. Unfortunately though even the Advisory Conciliation and Arbitration Service (2016) are no clearer as to how this legally permitted discrimination impacts LGBTs in actuality stating that:

The Equality Act has specific exemptions where employment is for the purpose of organized religion, such as being a Minister or otherwise promoting or representing the religion. This means that some roles can be restricted to people of a particular sexual orientation. There can also be additional requirements related to sexual orientation, such as a requirement for gay men or lesbians to be celibate. Restrictions concerning religion can also apply to the protected characteristics of sex, gender reassignment, and marriage and civil partnership.

It remains totally opaque as to what “promoting or representing the religion” infers in practice and given that the right to choose a partner is broadly protected especially under Article eight of the Human Rights Act 1998 “Right to respect for private and family life” the fact that additional requirements of religious organisations can require gay men or lesbians to be celibate is of concern and contra to human rights law. Denying a person sexual agency (as with discrimination in general) is also a form of psychological violence which could engender physical harm such as raised blood pressure and likely cardiovascular disease (Everett and Mollborne 2013)^9^ or even, suicide (Paul et al., 2011). Thus across the United Kingdom it appears to be the case that LGBTQ staff working for religious institutions can rightly fear being fired if their orientation comes to light whilst anyone revealing their sexual orientation as LGBTQ at an interview for work with a religious institution may fear being refused a job.^10^ In line with the views of Hay (1993) once employed, the fear of being sacked from the employ of a religious organization on grounds of sexual orientation coming to light or, being refused work/fearing refusal can all be considered as examples of law’s violence whereby this takes the form of psychological harm (not forgetting the long-term impact of psychological harm on the body biologically) and financial penalties/poverty/lack of opportunity to compete on equal grounds within (heteronormative) society (Moore, 2009)^11^ It may indeed be the case that these exemptions lend support to anti-LGBT stances taken by protestors wanting to appear both right [teous] and likeable, especially under the guize of religion. Despite this grave situation, the Government Equalities Office National LGBT Survey (2018: 5) claimed that:

Since 1967, when Parliament partially decriminalized male homosexual acts in England and Wales, the United Kingdom has made significant progress to advance equality for LGBT people. Recent milestones include bringing in the Marriage (Same-Sex Couples) Act 2013, which allowed same sex couples to marry, and introducing “Turing’s Law” in the Policing and Crime Act 2017, which posthumously pardons men who were convicted for having sex with men prior to 1967 where the offense is no longer a crime. Our Parliament now has the highest proportion of openly lesbian, gay and bisexual members of any legislature in the world and we are consistently ranked as one of the best countries in Europe for LGBT rights.

Nevertheless, the Government Equalities Office Survey (2018: 5) then cautioned that:

Despite this progress on legal entitlements, research and evidence has continued to suggest that LGBT people face discrimination, bullying and harassment in education, at work and on the streets, hate crime and higher inequalities in health satisfaction and outcomes.^12^

As redressed below, the issue of exemptions permitting discrimination against LGBTQs across the whole of the United Kingdom under equality laws may have emboldened Asher’s^13^ bakery in Northern Ireland (owned by evangelical Christians) to refuse to bake a cake with a slogan supporting gay weddings. These exemptions could also have encouraged religiously minded protestors to raise their voices outside a school in Birmingham in Spring 2019 discussed in due course. In the first instance, the Asher’s case is discussed.

In 2014 Gareth Lee “..had ordered a ‘..cake depicting the Sesame Street characters Bert and Ernie below the motto ‘Support gay marriage’ for an event to mark International Day Against Homophobia’ (McDonald, 2016) Thereafter Ashers refused to complete the order for the cake, declining to do so as it was ‘at odds with its beliefs” (BBC, 2019, A). Lee, supported by the Equality Commission for Northern Ireland took a case of discrimination out against the owners and won it. On appeal against the decision by the owners of Ashers in October 2016, the court upheld the original finding that Ashers had been guilty of discrimination. Following the decision, John O’Doherty director of the Rainbow Project, said:

Ashers Baking Company entered into a contractual agreement to make this cake and then changed their mind. Sympathetic as some may be to the position in which the company finds itself, this does not change the facts of the case. The judgment clearly articulated that this is direct discrimination for which there can be no justification (McDonald, 2016: NP).

At time of writing, the decision appears to refute the claim made here that exemptions within the equality legislation on grounds of sexual orientation may encourage discrimination yet as of August 2019 Ashers appealed to the European Court to have the decision overturned (BBC News, 2019). This is despite the fact that under 16.2. Two of the Equality Act Northern Ireland the following is stated: “2) This regulation [discrimination] does not apply 1) to an organization whose sole or main purpose is commercial”. It can be argued that Asher’s sole purpose was as a commercial enterprise and not religious. However, whether or not the exemptions within equality legislation (law’s violence) encouraged stances such as those taken by the owners of Asher’s Bakers, lines may be drawn between their objection to gay marriage, and, the impact of exemptions under the Equality Act 2010 in Britain and the protest against the inclusion of LGBT issues within the curriculum at a school in Birmingham in the United Kingdom in Spring 2019, broadcast widely by the media as discussed below.

Throughout the Spring of 2019 anger was shown by some members of the public toward inclusive education at a school in Birmingham England (Parveen, 2019). The loud disruptive demonstrations were concerned with, according to protestors, children being taught inappropriate content on LGBT issues. Parveen (2019) noted that:

Most of the protesters have been of Muslim faith and some have stood regularly outside the school chanting “Let kids be kids” and carrying placards with the message: “Adam and Eve, not Adam and Steve.”

Lines can be drawn here between the fact that Ashers are Christians and, most demonstrators in Birmingham were said to have been Muslim. It should not be overlooked however that Muslim demonstrators used the largely (yet not exclusively) Christian scenario of Adam and Eve to attract sympathy for their cause.^14^ However, Parveen (2019: np) also stated that:

Birmingham city council launched court action to prevent more protests outside the school after about 300 people gathered at the gates in May. The demonstration included a highly controversial speech by an imam who claimed [incorrectly that] anal sex, paedophilia and “transgenderism” were being taught in schools.

In November 2019 the High Court ruled that a temporary exclusion zone placed around the school to prevent protests would become permanent (Parveen, 2019) as:

…the anti-social behavior was having a “significant adverse impact” on pupils, teachers and the local community. Children at the school had to be kept inside with all the windows locked to avoid the “intolerable” noise from protesters on megaphones and often involving people with no direct connection to the school.

Delivering his verdict, Mr Justice Mark Warby said:

The judgment notes that the true position so far as the teaching is concerned has been misrepresented, sometimes grossly misrepresented, in the course of the protests. Speakers … have alleged that it [the school] is pursuing a “paedophile agenda”, and teaching children how to masturbate. None of this is true (Parveen, 2019).

Given the exemptions within equality legislation discussed earlier though, and, reflecting heteronormativity, it is of interest that:

…the Christian campaigner John Allman from Okehampton, Devon,….opposed the imposition of what he claimed would be a “super-injunction” and went on to score the only victory in the case when Justice Warby lifted his earlier ban on social media criticism of LGBT teaching, accepting the argument for free online speech (*ibid*: 2019).

Similarly to the Asher’s case, at the time of writing, following the verdict, the protesters said “an appeal was ‘highly likely’ and their campaign would go on with protests at the edge of the exclusion zone continuing” (Parveen, 2019). Overall then, the Ashers case and that of the protest in Birmingham are linked by objections based on religious grounds. Ashers refused to “support” gay marriage by advertising such a stance on a cake whilst the protestors in Birmingham appeared not to want children to hear about diversity. Both underscoring objections to any relationship that is not heteronormative underlined by (in the Birmingham case) placards related to the hugely symbolic scenario of Adam and Eve not “Adam and Steve”.

Given what has been stated so far all of us should be concerned that against this background hate crime against LGBTs is on the increase (Stonewall, 2019). A few years earlier in 2017 a survey by the TUC (2018) identified that “[A]round seven out of ten LGBT workers [had] experienced at least one type of sexual harassment at work (68 per cent)” therefore it appears to be the case that despite equality laws offering protection from discrimination, homophobia, underscored by the exemptions for religious beliefs within equality legislation (which likely act as a catalyst for discrimination) remains a very real problem (Rivers, 2011). Within such a hostile environment who knows how many children remain victims of a heteronormative agenda (Syracuse University, 2004) within families where any mention of LGBTQ issues may be met with silence or worse if they were to come out (Hockaday, 2019). In fact Hockaday (2019) indicated the worse-case scenario is the lived experience for some children: …research from the [Albert Kennedy Trust, a charity supporting LGBT youth] charity found that:

Twenty four per cent of homeless people aged 16–25 identified as LGBT+. More than three-quarters (77 per cent) citing abuse and rejection from their family as a reason for them losing their homes. A quarter of people surveyed by YouGov said relationships and sex education should not include LGBT issues, while 59 per cent supported including topics such as gender identity and sexuality at an age appropriate level

Thus a discussion is necessary as to the position of children and young people as they are educated (or not) as to the rights of LGBTQ people. The issue may also be perceived as a matter of health (especially mental health) and safety for all within all sectors of education in the United Kingdom, and, an issue regarding encouraging social cohesion. As exemplified earlier here, heteronormativity appears to loom large as underscored by the exemptions relevant to LGBTQs and religious organisations under equality laws. Currently, as advanced below, and perhaps reflecting exemptions for religious belief within equality legislation, it appears to be the case that largely within the United Kingdom there has been a failure to educate all students as to contemporary issues of diversity, potentially harming all in their future lives.

### LGBTQ Inclusion Within Currcula?

In recent times, possibly the greatest disservice done to the LGBTQ communities in the United Kingdom was the imposition of section 28 (Lee, 2019: 675) by Thatcher’s government whereby it was set out that:

A local authority shall not—1) intentionally promote homosexuality or publish material with the intention of promoting homosexuality; 2) promote the teaching in any maintained school of the acceptability of homosexuality as a pretended family relationship

Lee (2019: 676) comments that

State schools at that time in England were under local authority jurisdiction and so it was widely assumed that this piece of legislation prohibited LGBT + teachers from being open about their own sexual identity in the workplace, or discussing non-heterosexual relationships in their classrooms.

Despite the repeal of section 28 in 2003 (Twocock, 2019: np) inclusive education has yet to be addressed and as discussed by Lee (2019) the shadow cast by section 28 still impacts long-term on teachers who taught under that proviso. In September 2020 however it is said that ‘…new regulations for teaching relationships and sex education (RSE) in English schools would come into force (Twocock, 2019) so a new era is heralded for inclusivity. Yet it is doubtful that those who are homophobic will necessarily change their attitudes overnight and the exemptions outlined earlier can only bolster such prejudice. Therefore what is known about the experiences of gay young people in Britain’s schools and other areas of education remains of concern.

On reflection as to the “The School Report: the experiences of gay young people in Britain’s schools in 2012” (Guasp, 2012) in an online blog, Stonewall observed that:

…one in three lesbian, gay or bi young people who are bullied [at school] consider changing their future educational plans because of it, for instance by deciding not to go to university or college. Universities that take steps to combat homophobic, biphobic and transphobic bullying, and promote their work in this area, will encourage these young people to carry on in education and to apply to study at their institution (Stonewall, 2017)

Later, The National Union of Students (2014): 1) identified that:

…many LGBT people continue to feel isolated in education and society. Many suffer mental health and financial issues and all too often we hear cases of LGBT students leaving education as an indirect result of their identity.

In fact, many of the statistical results presented by the NUS in 2014 were cause for alarm: only two in ten trans students felt completely safe on campus being less than half the proportion of their heterosexual counterparts (43 per cent) whilst only 36.7% per cent of LGB + students felt completely safe on campus. One out of five LGB+ and one in three trans participants had experienced bullying (Mayo, 2013) or harassment on campus and LGB + students are reported as being slightly more likely to drop out of HE^15^ than heterosexuals:^16^ the rates are 25 per cent for heterosexual students, 27.7 per cent for gays, 26.6 per cent for lesbians and 30 per cent for bisexuals. In addition, more than half of LGB + respondents (56 per cent) cited that they felt they did not fit in as the main reason for dropping out. Moreover, it is reported that LGBT students having experienced homophobic or transphobic harassment are two—three times more likely to consider leaving their course. In addition 51% of trans students had considered dropping out of studies.

The perception of LGBTQ students not fitting in and subsequently dropping out of university underscores the need for LGBTQs to see themselves reflected much earlier within curricula (Taylor, 2020). In fact, on the issue of primary school, where inclusive education could help to embed the ethos of respect for diversity in all children at a much earlier age Taylor (2020: np) observes that:

Children are presented with a range of narratives, stories and movies featuring heterosexual relationships from a very young age, so it’s vital for them to see same-sex relationships too, otherwise we are telling them that these relationships are different, “other” and in some way shameful.

Taylor's (2020) observations aside though, within HE it appears to be the case that with regards actual pedagogic practice, on a scale of 1–10 it was reported that:

LGB + students’ average score of agreement with the statement “I see LGB experiences and history reflected in my curriculum” is only 3.9 and for trans students it is 3.5. For the statement, “I see trans experiences and history reflected in my curriculum,” the scores are 2.8 for LGB + students and 2.5 for trans students^17^ (National Union of Students, 2014: 2).

Perhaps not surprisingly it was found by the National Union of Students, (2014) that “LGBT students who are out to their tutors tend to feel more confident to speak up in class (89 per cent) than those who are only out to their friends (79 per cent)”^18^ (*ibid:* 6). However, it is also stated that “Gay men students tend to experience more harassment and abuse on the grounds of their sexual orientation than lesbian and bisexual women” (National Union Students, 2014: 24). So in 2014 it was evident that there was room for improvement regarding the situation of LGBTQ students.

Given the issues raised above, concerns remain as to the situation of any LGBT students that consolidate their educational journey by undertaking studies in Higher Educational institutions. In fact, underscoring these concerns, in 2017 Gale and Ward (2017a) warned that whilst: “In the United Kingdom, we have made great legal strides toward equality”…”culturally we are still playing catch up—and higher education is just one of the places where LGBTQ people are being failed.”

More lately, in another report for Stonewall (Bachman and Gooch, 2018: 3) Ruth Hunt Chief Executive stated that:

University is a time for all students to learn, grow and enjoy independence. But for many lesbian, gay, bi and trans students, the experience can be marred by discrimination, exclusion and abuse because of who they are.

Despite Hunt stating that progress has been made regarding equality and inclusion for LGBTQs on campuses across the United Kingdom it is of concern that the following issue was identified:

University remains a challenging environment for many LGBT students to be themselves. A concerning number of LGBT students still don’t feel safe to disclose their sexual orientation and/or gender identity. Some have even been encouraged by university staff to hide their identity at university (Bachman and Gooch, 2018: 9).

The importance of the above finding is underscored by the fact that:

A welcoming university environment is important because many LGBT students cannot be open about being LGBT with their family. One in five lesbian, gay and bi students (20 per cent) aren’t open to anyone in their family about their sexual orientation. One in six trans students (16 per cent) aren’t open to anyone in their family about their gender identity (*ibid*: 9).

Perhaps of utmost concern, given their intersectionality, was the finding that: ‘LGBT disabled students are particularly likely to have been the target of such remarks from other students; almost half of LGBT disabled students (47 per cent) have experienced this (Bachman and Gooch, 2018: 9). However, the fact that “[M]ore than two in five LGBT students (42 per cent) hid or disguised that they are LGBT at university in the last year because they were afraid of discrimination” appears in stark contrast to the progress on LGBTQ issues claimed by Hunt (Bachman and Gooch, 2018: 3). Perhaps reflecting a swing to the right in society, if students are hiding their orientation we cannot know the true state of play regarding discrimination. There are also other concepts relevant to an inquiry into the experiences of LGBTQs within HE that should be noted. For example, the impact of minority stress which can encompass some or all of the following issues: feeling unsafe on campus especially in student accommodation, the reported higher drop-out rate of LGBTQ students, issues concerning mental health, lack of parental support for LGBTQ students in some cases, some staff lacking confidence to challenge homophobia, and, a higher suicide rate for members of these communities (Gale and Ward, 2017b: B). Overall then, exemptions permitting legal discrimination within equality legislation appear very harmful indeed when combined with the knowledge that many children and young adults are being let down with the education system as the majority are uninformed as to diversity within society.

## Conclusion

The concept of heteronormativity was raised briefly to underscore the probable rationale for discrimination (when it occurs) against LGBTQs in society. Heteronormativity being “an ideology based on definitions of what it means to be a woman or a man that exclude and discriminate against a significant minority population.” (Syracuse University, 2004: iv). Allowing age old revered and outmoded beliefs to trump rights may be one of the major reasons why exemptions exist relative to equality legislation across the United Kingdom, underscored by the infamous Asher’s cake row incident, and, perhaps influencing protestors outside a school in Birmingham in 2019. However, whilst within many jurisdictions such heteronormative stances are starting to be challenged and redressed all students of all ages are impacted by discrimination as children and young people are failed in the United Kingdom by an education system that has largely overlooked diversity in education concerning same-sex relationships. Yet hopefully that will change in due course (Twocock, 2019). Meanwhile though even at HE level, many concerns remain as to inclusion and diversity within the curriculum for LGBTQ students (whilst the impact on LGBTQ staff must not be overlooked) and students of all ages continue to be let down. Overall, I believe that religious organisations should not be permitted to legally discriminate against LGBTQ people in any form and that this top down discrimination permitted within *equality* law sends out entirely the wrong message emboldening some homophobic religious zealots within society to believe themselves right [teous] and likeable when their homophobia is permitted and encouraged by exemptions that turn equality law into the opposite of what, in this particular case, it was legislated for: to counteract the violence of homophobia be that experienced physically or psychologically/emotionally or even in the form of poverty (for example when an LGBTQ person loses their job in a religious organisation/fails to be employed on those grounds). Therefore at present across society in the United Kingdom LGBTQs are left in no doubt they are not fully equal to their heterosexual counterparts within United Kingdom law. And this must change. Amen.

## Footnotes

2
https://www.equalityhumanrights.com/en/equality-act/protected-characteristics.

3 See the chapter entitled “Criminalizing Queerness” for Buist and Lenning’s (2016) discussion on the impact of anti-LGBT legislation in the United States in Queer Criminology, Routledge.

4 See Gov.uk: https://www.gov.uk/discrimination-your-rights/how-you-can-be-discriminated-against [Accessed: 14/10/20].

5 Prior to the Act being passed Petre (2006) revealed it was clear some religious leaders voiced objections for example: Rupert Kaye, the chief executive of the Association of Christian Teachers, said: “Diverse individuals and organisations should be free to agree to disagree. They should not be required by law to show ‘mutual respect’ to individuals or organisations whose beliefs or lifestyle are anathema.” Whilst “Dr. Majid Katme, the spokesman for the Islamic Medical Association, argued that the proposals demonstrated that the Government was prepared to discriminate against faith communities in order to promote ‘equality’.”

6 Square brackets denote my own views.

7
http://www.legislation.gov.uk/nisr/2006/439/regulation/16/made.

8 See also the following: Circumstances when being treated differently due to sexual orientation is lawful: A difference in treatment may be lawful if:…..a religious or belief organization is excluding persons of a particular sexual orientation from its membership or participation in its activities, or its provision of goods, facilities and services. This only applies to organisations whose purpose is to practice, promote or teach a religion or belief, whose sole or main purpose is not commercial. The restrictions they impose must be necessary either to comply with the doctrine of the organization, or to avoid conflict with the “strongly held religious convictions” of the religion’s followers. https://www.equalityhumanrights.com/en/advice-and-guidance/sexual-orientation-discrimination [Accessed: 12/10/20].

9 Everett and Mollborne (2014: 1) found that: Gay or mostly gay men face almost twice the risk of being hypertensive compared to heterosexual men. And that “Increased exposure to stigma and discrimination may in part explain gay men’s increased cardiovascular risk.”

10 I have met people who work for religious organisations in the United Kingdom and live in fear of being outed and losing their jobs. I also know an academic colleague who was refused a job with a religious organization because she came out to the interviewer.

11 In discussion of the Act that was soon to become law Moore (2009) stated that: “A Government Equalities Office spokesman said: ‘The Equality Bill will not force a church to accept someone as a priest regardless of their sexual orientation or gender.’” Also that: “Churches, synagogues, mosques and others will continue to have the freedom to choose who they employ in jobs which promote their religion. But where they provide services to the public they will have to treat everyone fairly.”

12 The same survey also noted that a quarter (24%) of all respondents living with family members, excluding partners, were not open at all about being LGBT (2018:12).

13 Ashers “gay cake” row referred to European Court https://www.bbc.co.uk/news/uk-northern-ireland-49350891#.

14 The author’s opinion is that doing so was used to elicit greater sympathy and support from religious others across the United Kingdom.

15 As indicated by the Albert Kennedy Trust later in this article many homeless youths are found to be LGBTQ. It is reported that when some students come out to their parents, some are then cut off from financial and emotional support. This issue is under-researched and it may be the case that LGBTQ students are far *more* likely to drop out of education due to this type of scenario.

16 There is a gap in information here: what about those students that are not open about their orientation/those that were not consulted or declined to engage with the survey.

17 NUS (2014: 6).

18 Many other authors have also influenced this research proposal with work concerning queer pedagogy, for example: Bryson and Castell (1993) “Queer Pedagogy; Praxis Makes I'm/Perfect,” Canadian Journal of Education/Revue canadienne de l'èducation, Vol. 18, No. 3, Against the Grain: Narratives of Resistance, (Summer, 1993), pp. 285–305; Canon and Linhorst (2007) How will they understand if we don't teach them?: The status of criminal justice education on gay and lesbian issues; Dwyer (2015) “Teaching Young Queers a Lesson: How Police Teach Lessons about Non-Heteronormativity in Public Spaces,” Sexuality and Culture, September 2015, Volume 19, Issue 3, pp 493–512; The Global Alliance for LGBT Education (2017) “The USA education secretary sees no problem when schools refuse LGBT students” Available at: https://www.gale.info/en/news/local_news/170525-devos-ok-with-discrimination-lgbt-students (Accessed: 05/07/17); Ren, A. K (2010) “LGBT and Queer Research in Higher Education: The State and Status of the Field,” Educational Researcher, Vol. 39, No. 2, pp. 132–141 Available at: https://msu.edu/∼renn/RennLGBTQueerEdResearcher.pdf; Lecky, R. and Brooks. K. (2010) Queer Theory: Law, Culture, Empire, Routledge, Glasshouse Books, Oxon.
